# Facilitators and barriers to acceptance of coronary artery bypass graft surgery: a qualitative study

**DOI:** 10.1186/s13019-025-03806-y

**Published:** 2025-12-29

**Authors:** Sajjad Ebrahimi, Efat Sadeghian, Masoud Khodaveisi, Arash Khalili, Tayebeh Hasan Tehrani

**Affiliations:** 1 School of Nursing and Midwifery, Hamadan University of Medical Sciences, Hamadan, Iran; 2 Chronic Diseases (Home Care) Research Center, Institute of Cancer, Hamadan University of Medical Sciences, Hamadan, Iran; 3 Department of Nursing, School of Nursing and Midwifery, Hamadan University of Medical Sciences, Hamadan, Iran; 4 Department of Community Health Nursing, School of Nursing and Midwifery, Hamadan University of Medical Sciences, Hamadan, Iran; 5 Mother and Child Care Research Center, Institute of Health Sciences and Technologies, Hamadan University of Medical Sciences, Hamadan, Iran; 6 Department of Pediatric Nursing, School of Nursing and Midwifery, Hamadan University of Medical Sciences, Hamadan, Iran

**Keywords:** Coronary artery bypass, Social support, Trust, Decision making, Culture

## Abstract

**Objective:**

To elucidate facilitators and barriers shaping Iranian patients’ decisions to accept or avoid coronary artery bypass graft (CABG) surgery, focusing on psychological, social, and cultural influences.

**Methods:**

This 2024 qualitative study used conventional content analysis with purposive sampling at Bushehr Heart Hospital, Iran. Twenty-four semi-structured interviews were conducted with 18 participants (11 patients, 2 family members, 2 nurses, 3 physicians). Data were analyzed inductively using MAXQDA20 software.

**Results:**

Eight categories emerged from 24 subcategories. Facilitators of CABG acceptance included Trust in Healthcare Providers, Treatment Transparency, and Infrastructure; Social and Family Support; Access to Information and Specialist Medical Consultation; Learning from Others’ Experiences; and Psychological and Spiritual Coping. Barriers included Fear of Surgical Outcomes; Preference for Major City Hospitals; and Professional Challenges. Trust, strong social support, and positive peer experiences fostered acceptance, while fears, preference for major city hospitals, and professional challenges posed barriers.

**Conclusion:**

Clear communication, social support, and culturally sensitive counseling enhance CABG acceptance. These insights, grounded in Iran’s context, inform global strategies to optimize patient decision-making and postoperative outcomes.

## Introduction

Cardiovascular diseases (CVDs) remain a leading cause of mortality worldwide, responsible for over 20 million deaths in 2021 [1]. In Iran, CVDs caused 236,000 deaths in 2013, with a 49% risk for men and 32.5% for women developing CVDs after age 40 [2]. Coronary artery bypass graft (CABG) surgery is a cornerstone treatment for severe coronary artery disease when medication or less invasive interventions are insufficient [3]. Globally, over one million CABG procedures are performed annually [4, 5].

The decision to undergo CABG is complex, influenced by psychological, social, cultural, and economic factors. In Western settings, fear, inadequate health literacy, and financial barriers are well-documented drivers of surgical hesitation [6, 7]. In Iran, centralization culture, defined as preference for major city hospitals like Tehran or Shiraz for perceived superior expertise, may amplify these challenges, yet qualitative research exploring these dynamics remains limited [8, 9]. Such factors can lead to delays or avoidance of surgery, adversely affecting mental health and postoperative quality of life [10, 11]. Qualitative methods, exploring lived experiences, are ideal for capturing these nuances [12].

This study aims to elucidate the factors influencing Iranian patients’ acceptance or avoidance of CABG surgery, examining how centralization culture, family-driven decisions, and professional challenges interact with psychological and informational factors in informed decision-making, defined as weighing risks and benefits with adequate information [13–15]. By identifying these, healthcare teams and policymakers can develop targeted preoperative counseling to improve surgical uptake and postoperative quality of life in Iran and globally.

## Methods

This qualitative study employed conventional content analysis to explore factors influencing Iranian patients’ acceptance or avoidance of CABG surgery, allowing categories to emerge inductively from participant experiences [12]. This approach was chosen to capture cultural and psychosocial nuances underlying decision-making.

### Participants and recruitment

The study was conducted in 2024 at Bushehr Heart Hospital, a specialized CABG center in southern Iran. Using purposive sampling, 18 participants (10 men, 8 women; aged 41–78 years) with diverse educational and ethnic backgrounds (Persian, Arab, and others) were recruited. The sample included CABG candidates, post-surgery patients, two family members, two nurses, and three physicians (two cardiac surgeons, one cardiologist) to ensure a range of perspectives.

Data saturation occurred after 24 interviews when no new categories emerged. The last four interviews were used to confirm and deepen category definitions. Six follow-up interviews clarified ambiguities, during which no novel concepts emerged and previous data were repeated; thus, data saturation was confirmed. Iterative coding and team consensus preserved analytical clarity throughout.

### Inclusion and exclusion criteria

Participants were eligible if fluent in Persian, able to communicate, and willing to share their CABG-related experiences. Patients represented different decision stages (acceptance, hesitation, refusal) and were within three months post-surgery. Although the majority of patient participants had accepted surgery, potentially underrepresenting avoidance perspectives, this was addressed by including hesitant patients and family members. Individuals unable to complete interviews due to medical emergencies or who withdrew consent were excluded.

**Table 1:** Demographic characteristics of participants

Group	Characteristic	Details
**Patients**	Gender	6 males, 5 females
	Age	41–78 years (mean 63 years)
	Marital Status	9 married, 1 single, 1 divorced
	Education Level	Primary to bachelor’s degree
	Length of Stay	7–30 days
	Number of Admissions	1–6 times
	Ward	8 ICU, 4 Angiography, 5 Post-Open-Heart Surgery, 7 CCU
**Family**	Gender	1 male, 1 female
	Age	31–48 years
	Education	Diploma to bachelor’s degree
	Occupation	Employee, housewife
	Marital Status	1 married, 1 single
**Nurses**	Gender	2 females
	Age	42–44 years
	Education	Bachelor’s degree
	Work Experience	16–18 years
	Marital Status	Married
**Doctors**	Gender	3 males
	Role	2 cardiac surgeons, 1 cardiologist
	Age	48–59 years
	Work Experience	14–23 years
	Marital Status	Married

### Data collection

A total of 24 semi-structured face-to-face interviews were conducted in private hospital rooms or staff educational spaces, each lasting 35–50 min. Written informed consent was obtained, and all sessions were audio-recorded with permission. Field notes captured contextual and nonverbal observations.

Interviews began with open-ended prompts such as “Can you describe your heart condition journey?” and included key questions like “What factors influenced your decision to accept or avoid CABG?” and “How did family support affect your decision?” — the latter being instrumental in shaping the Social and Family Support code. The full interview guide is provided in Appendix A.

### Data analysis

All interviews were transcribed verbatim in Persian and analyzed using MAXQDA20 software. The process followed the conventional content analysis framework by Hsieh and Shannon [16] and Erlingsson and Brysiewicz [17]. Meaning units were identified and inductively coded, then merged into 24 subcategories and 8 categories representing facilitators and barriers (Table 2). For example, *“the doctor explained everything clearly”* was coded as *“transparent communication”* under *“Trust in Healthcare Providers*,* Treatment Transparency*,* and Infrastructure.”* The primary analyst conducted initial coding, and 20% of transcripts were double-checked by another researcher. Coding decisions were refined through team discussions and validated with five participants during member-checking.

**Table 2:** Categories and subcategories derived from data analysis

Category	Subcategories	Type (Facilitator/Barrier)
Trust in Healthcare Providers, Treatment Transparency, and Infrastructure	• Medical team expertise and skills • Timely, comprehensive information • Transparent communication • Modern, high-quality equipment • Hygiene and safety standards • Supportive, calming hospital environment	Facilitator
Social and Family Support	• Continuous family presence • Family involvement in treatment decisions • Emotional and psychological support	Facilitator
Access to Information and Specialist Medical Consultation	• Consultations with multiple physicians • Understanding treatment risks and benefits • Comparing physicians’ expertise and opinions	Facilitator
Learning from Treatment Experiences of Others	• Positive feedback from other patients • Observing postoperative challenges in others	Facilitator (positive)/Barrier (negative)
Psychological-Spiritual Coping Mechanisms	• Relaxation techniques (e.g., deep breathing) • Spiritual beliefs and reliance on faith	Facilitator
Fear of Surgical Outcomes	• Fear of surgical complications • Fear of death • Fear of work loss • Fear of body image changes	Barrier
Centralization Culture (Preference for Major City Hospitals)	• Preference for major city hospitals • Belief in superior care at centralized hospitals	Barrier
Professional Challenges	• Conflicting medical opinions • Inadequate or contradictory information	Barrier

### Rigor (Trustworthiness)

Rigor was established following Lincoln and Guba’s criteria [18, 19]. Credibility was enhanced by prolonged engagement and member-checking with patients, relatives, and clinical staff. Dependability was supported through double-coding and external review of the codebook. Confirmability was maintained using reflexive notes, coding memos, and an audit trail of analytic decisions. Transferability was strengthened by diverse sampling and comparison with four non-participating patients. All researchers had formal training in qualitative research, and transparency was ensured through the 32-item COREQ checklist (Appendix B).

### Ethical considerations

This study, part of Hamadan University of Medical Sciences project (14029147954), was approved by its Ethics Committee (IR.UMSHA.REC.1402.073). Participants were informed about objectives, procedures, and audio recording. Participation was voluntary, with confidentiality assured. Participants could withdraw or access transcripts. Recordings and transcripts were stored on a secure, password-protected server.

## Results

Interconnections among categories were evident, such as trust in healthcare providers mitigating fears of surgical outcomes, social support enhancing access to information, positive peer experiences fostering hope, and spiritual coping reducing anxiety, reflecting the complex interplay of psychological, social, and cultural factors in decision-making. Eighteen participants (10 men, 8 women, mean age 56.4 ± 10.6 years) contributed through 24 interviews (Table 1). The analysis yielded 640 initial codes, which through continuous comparison were distilled into 24 subcategories, and further comparison resulted in 8 categories, grouped into facilitators and barriers (Table 2).

### Facilitators of CABG acceptance

These categories empowered patients to navigate uncertainties, fostering confidence and enabling informed surgical decisions.

### Trust in healthcare providers, treatment transparency, and infrastructure

Drawing from six subcategories—medical team expertise and skills; timely, comprehensive information; transparent communication; modern, high-quality equipment; hygiene and safety standards; and supportive, calming hospital environment—initial codes highlighted elements like “surgeon’s years of experience” and “detailed pre-op explanations reducing uncertainty.” Medical team expertise and skills featured codes such as “doctor’s successful track record in CABG cases,” while timely and comprehensive information involved codes on “receiving full details on procedure steps.” Transparent communication reflected codes like “honest discussions about success rates,” and modern equipment stemmed from codes such as “availability of advanced monitoring tools.” Hygiene and safety standards included codes on “sterile wards preventing infections,” with the supportive environment incorporating codes like “empathetic staff interactions.” As one participant reflected, *“The doctor’s clear explanation made me trust the process” (P12)*, and a 55-year-old female added, *“The hospital’s advanced equipment and clean wards reassured me” (P8)*. This categorie, driven by skilled medical teams, transparent communication, advanced technology, and clean hospital environments, significantly bolstered patient confidence. Participants valued clear explanations from physicians and reliable facilities. A 48-year-old male noted: *“The doctor’s clear explanation and the surgeon’s experience gave me peace of mind” (P12)*. A 63-year-old male shared: *“Advanced technology and a clean hospital reassured me” (P14).* Another emphasized: *“The hospital’s hygiene and staff professionalism built my trust” (P7).*

### Social and family support

Encompassing three subcategories—continuous family presence; family involvement in treatment decisions; and emotional and psychological support—initial codes captured elements such as “family accompaniment during consultations” and “shared emotional burden in crisis moments.” Continuous family presence was evident in codes like “relatives staying bedside post-diagnosis,” while family involvement in decisions drew from codes such as “joint review of surgical options.” Emotional and psychological support arose from codes on “encouragement reducing fear,” with a patient noting, *“My family researched the surgery and gave me courage” (P7)*, and a 60-year-old male adding, *“My son consulted multiple doctors to ensure I made the right choice” (P10)*. Family and friends provided emotional and practical support, acting as a vital buffer against stress and encouraging surgical acceptance. Family involvement in decision-making and presence during crises were crucial. A 68-year-old patient shared: *“My family’s support gave me confidence to proceed” (P16).*

### Access to information and specialist medical consultation

Incorporating three subcategories—consultations with multiple physicians; understanding treatment risks and benefits; and comparing physicians’ expertise and opinions—initial codes included “seeking second opinions” and “weighing pros/cons lists from doctors.” Consultations with multiple physicians involved codes like “visiting specialists for confirmation,” while understanding risks and benefits featured codes such as “detailed breakdowns of mortality rates vs. recovery chances.” Comparing expertise was based on codes like “weighing specialists’ opinions on surgery versus angioplasty,” as a 62-year-old patient explained, *“Consulting specialists clarified risks*,* but differing opinions made me hesitate” (P4)*. Consultations with specialists and detailed risk-benefit discussions empowered patients to make informed choices. Conflicting opinions, however, occasionally caused hesitation. A 60-year-old patient explained: *“After consulting several doctors*,* I felt informed enough to choose surgery” (P7).* Another highlighted: *“Understanding the risks and benefits clarified my decision” (P3).*

### Learning from positive treatment experiences of others

Built upon two subcategories—positive feedback from other patients; and observing managed postoperative challenges—initial codes encompassed “hearing success stories” and “witnessing quick recoveries in wards.” Positive feedback from other patients derived from codes like “peers sharing low pain levels post-op,” while observing managed challenges highlighted codes on “learning from successfully managed complications,” with a 50-year-old female stating, *“Hearing about my neighbor’s recovery gave me confidence” (P5)*. Positive peer experiences inspired hope and reinforced confidence in successful outcomes. A 62-year-old stated: *“Seeing patients recover gave me hope for my own surgery” (P12).*

### Psychological and spiritual coping mechanisms

Composed of two subcategories—relaxation techniques (e.g., deep breathing); and spiritual beliefs and reliance on faith—initial codes featured “personal rituals for calm” and “prayer as anxiety reducer.” Relaxation techniques included codes such as “breathing exercises before consultations,” while spiritual beliefs drew from codes like “invoking divine support for strength.” Relaxation techniques and spiritual beliefs helped patients manage anxiety, enabling calmer decisions. Faith-based practices provided comfort. A 55-year-old female said: *“Praying gave me strength to face surgery” (P9).* Another shared: *“Deep breathing calmed my nerves before deciding” (P4).*

### Barriers to CABG acceptance

These factors heightened hesitation, often leading to delays or refusal of surgery.

### Fear of surgical outcomes

Emerging from four subcategories—fear of surgical complications; fear of death; fear of work loss; and fear of body image changes—initial codes covered “post-op infection worries,” “mortality statistics overwhelming patients,” “job absence impacts,” and “visible scars affecting self-esteem.” Fear of complications was based on codes like “hearing about rare failures,” while fear of death included codes such as “mortality statistics.” Fear of work loss drew from codes like “job absence impacts,” and fear of body image changes included codes on “visible scars.” Concerns about complications, mortality, work disruption, and body image changes, particularly among women and elderly patients, hindered decision-making. A 54-year-old female expressed: *“I fear dying or looking different” (P6)*. A 65-year-old male added: *“I worried about losing my job if recovery took too long” (P11).*

### Centralization culture

Rooted in two subcategories—preference for major city hospitals; and belief in superior care at centralized hospitals—initial codes included “rumors of Tehran expertise” and “local facility distrust.” Preference for major cities stemmed from codes like “family advice to travel,” while belief in superior care involved codes on “assumed better tech in the capital.” Societal preferences for major city hospitals created doubts about local facilities’ capabilities. Clear explanations from local providers, however, mitigated concerns. A 60-year-old male stated: *“Everyone said big cities like Tehran have better doctors” (P9).* A 52-year-old female noted: *“I doubted our hospital until the surgeon explained its capabilities” (P11).* Another emphasized: *“I thought only Tehran hospitals were safe” (P13).*

### Professional challenges

Consisting of two subcategories—conflicting medical opinions; and inadequate or contradictory information—initial codes highlighted “differing doctor recommendations” and “confusing online vs. clinical info.” Conflicting opinions arose from codes like “one doctor advises surgery, another waits,” while inadequate information included codes on “rushed consultations lacking details.” Conflicting medical opinions and contradictory information undermined trust, delaying decisions. A 55-year-old male noted: *“Doctors’ disagreements confused me*,* making trust difficult” (P10)*. A 57-year-old female added: *“Different doctors gave conflicting advice*,* which scared me” (P2).*

## Discussion

Findings from this study explore facilitators and barriers shaping Iranian patients’ decisions to accept or avoid CABG surgery at Bushehr Heart Hospital in 2024, offering insights for culturally sensitive cardiothoracic care [20]. Eight categories—Trust in Healthcare Providers, Treatment Transparency, and Infrastructure; Social and Family Support; Access to Information and Specialist Medical Consultation; Learning from Others’ Experiences; Psychological-Spiritual Coping; Fear of Surgical Outcomes; Centralization Culture; and Professional Challenges—illuminate the intricate interplay of psychological, social, and cultural influences on patient choices.

### Facilitators of CABG acceptance

Findings from this study show that Trust in Healthcare Providers, Treatment Transparency, and Infrastructure emerged as a key facilitator, with patients valuing clear communication, skilled medical teams, and modern facilities. Analysis of this category indicates how expertise, transparency, and hygiene foster patient confidence, as evidenced by codes emphasizing detailed explanations and safe environments. Resonating with Arikan et al. (2024), who highlight that transparent communication builds confidence [20]. Patients frequently emphasized the role of experienced surgeons and hygienic environments in alleviating concerns. The findings suggest that hurried interactions, often due to high patient volumes in Iran’s public hospitals, erode trust and delay decisions, per codes on rushed consultations. Dawod et al. (2024) advocate structured preoperative counseling to deliver consistent, patient-centered information, a strategy vital for Iran’s busy healthcare settings [21]. Training surgical teams in empathetic, clear communication could address these barriers effectively, fostering trust in high-pressure environments.

Findings from this study indicate that Social and Family Support played a vital role, with families providing emotional and decision-making support to alleviate stress. Family involvement and emotional backing in this category ease uncertainty in collectivist contexts, illustrated by codes on joint option reviews and fear reduction. Aligning with van Dieën et al. (2024), who emphasize family involvement in navigating surgical uncertainty [7]. In Iran’s collectivist culture, family members often act as primary decision-makers, gathering information and coordinating care. Shared burdens in crisis moments, as captured by codes, underscore this dynamic. Engaging families in preoperative discussions could enhance their role. Access to Information and Specialist Medical Consultation empowered patients, though conflicting advice caused confusion. Consultations and risk assessments within this category clarify options yet induce hesitation through differing views, as shown in codes on mortality breakdowns and opinion variances. Arikan et al. (2024) recommend coordinated counseling, suitable for Iran [20]. Learning from Others’ Experiences fostered hope through positive peer stories. Peer feedback in this category alleviates anxiety via success narratives, per relevant codes. Zaidova et al. (2022) suggest peer support programs to highlight successful CABG outcomes, which could reduce anxiety among Iranian patients [6]. Psychological-Spiritual Coping, encompassing relaxation techniques and spiritual beliefs, managed stress. Coping strategies in local contexts emerge through codes on breathing exercises and prayer for anxiety relief. Consistent with Sarhadi et al. (2024) [22].

### Barriers of CABG acceptance

Findings from this study highlight that Fear of Surgical Outcomes, especially among women and elderly patients, posed a significant barrier, driven by concerns about complications, mortality, or body image. Societal pressures and recovery fears in vulnerable groups are underscored by this category, via codes on mortality data and visible scarring. Arikan et al. (2024) propose targeted counseling to address these fears, emphasizing recovery timelines [20]. Women often cited societal pressures about physical appearance, while elderly patients worried about prolonged recovery. Centralization Culture, reflecting preferences for major city hospitals, presented unique challenges, with patients favoring centers in Tehran or Shiraz for perceived superior expertise. This category traces disparities in infrastructure perceptions, from codes on expertise rumors and assumed advanced tech. Echoing Hammami et al. (2020) [23]. In Iran, this preference often stemmed from perceptions of better doctors and technology in urban centers, rooted in historical disparities in healthcare infrastructure. However, clear communication from local providers about hospital capabilities reduced this bias, as seen in patients who gained confidence in Bushehr Heart Hospital after detailed explanations. Codes on local distrust emphasize campaigns for equitable access. These findings highlight the need for public awareness campaigns to promote trust in local facilities, ensuring equitable access to care. Unlike decentralized healthcare systems in Europe, where local hospitals are often trusted, Iran’s centralized culture poses unique challenges, underscoring the importance of culturally sensitive interventions.

Professional Challenges, including conflicting medical opinions and inadequate information, compounded barriers, eroding trust, and complicating decisions. This category captures systemic time constraints, as reflected in codes on rushed consultations by surgeons. A surgeon noted: *“Time constraints sometimes hinder clear explanations*,*”* aligning with Dawod et al. (2024), who suggest structured checklists to improve preoperative counseling [21]. In Iran’s overburdened healthcare system, time pressures often limit patient-provider interactions, exacerbating confusion.

This analysis, derived from 640 initial codes condensed into 24 subcategories and eight categories, revealed how trust, fear, and social support dynamically interacted to shape patients’ decision-making. Findings from this study, based on analysis of 640 codes and 24 subcategories, align with the Health Belief Model, where trust, family support, and peer experiences enhance perceived benefits, while fears, centralization culture, and professional challenges intensify barriers. Structured preoperative counseling sessions, peer support groups, and communication workshops can address these. Limitations include the qualitative design’s limited generalizability due to its single-center focus at Bushehr Heart Hospital in southern Iran, potentially restricting the applicability of findings to other regions. Additionally, the exclusion of patients unable to complete interviews due to medical emergencies or those withdrawing consent introduces selection bias. Cultural limitations also arise, as the study reflects the specific sociocultural context of southern Iran, which may not fully capture diverse cultural attitudes across the country. Future research should explore diverse urban contexts and other cities, testing interventions like mobile apps for education to address fears and cultural biases.

## Conclusion

This study underscores the complex interplay of trust, social support, and contextual influences shaping Iranian patients’ decisions to accept or avoid coronary artery bypass graft (CABG) surgery at Bushehr Heart Hospital. Trust in healthcare providers, fostered through clear communication to address conflicting medical opinions and modern facilities, mitigates fears and builds patient confidence. Social and family support, exemplified by families researching options and offering emotional encouragement, empowers patients to navigate surgical uncertainty, as seen in patients’ accounts of family-driven decisions. Spiritual beliefs and peer experiences further facilitate coping, while cultural barriers, such as a preference for hospitals in major cities like Tehran or Shiraz, and psychological fears about surgical outcomes, particularly among women and elderly patients, require targeted interventions. These insights from Iran’s context inform global cardiothoracic practice. Preoperative counseling programs tailored to address fears and cultural biases, alongside peer support initiatives showcasing successful outcomes, can enhance patient confidence across diverse settings, from Iran’s centralized healthcare system to decentralized models elsewhere. By integrating trust-building strategies, family engagement, and culturally sensitive care, healthcare teams can optimize decision-making, improve surgical uptake, and enhance postoperative quality of life for patients worldwide.

## Data Availability

No datasets were generated or analysed during the current study.

## Appendix A: interview guide

### Purpose

This guide was designed to explore factors influencing patients’ decisions to accept or avoid coronary artery bypass graft (CABG) surgery, focusing on psychological, social, cultural, and informational aspects.

- **Opening Question**: “Please describe your heart condition journey.”
- **Core Questions**:
  - “What factors influenced your decision to accept or avoid CABG?”
  - “How did trust in doctors or the hospital environment affect your choice?”
  - “What role did family or social support play?”
  - “Describe any fears or concerns about the surgery.”
  - “How did others’ experiences influence you?”
  - “How did perceptions of hospitals in major cities like Shiraz or Tehran versus local hospitals influence your decision?”
- **Probes**:
  - “Can you elaborate on that?”
  - “What specific information helped or confused you?”
  - “Can you describe a specific experience that shaped your decision?”

## Appendix B: COREQ checklist (32 Items)

**Table:**

Domain/Item	Description/Location in Manuscript
**Domain 1: Research Team and Reflexivity**	
**1. Interviewer/facilitator**	SE and THT conducted interviews (Methods 2.3)
**2. Credentials**	Ph.D. student (SE), faculty members (THT, ES, MKh, AKh) (Author Information)
**3. Occupation**	Nursing, pediatric nursing, community health nursing (Author Information)
**4. Gender**	Mixed-gender team (not specified per participant) (Methods 2.1)
**5. Experience and training**	Team trained in qualitative methods through formal courses and prior studies at Hamadan University (Methods 2.5)
**6. Relationship established**	No prior relationship with participants; rapport built during interviews via open-ended questions (Methods 2.3)
**7. Participant knowledge of the interviewer**	Participants informed about researchers’ roles and study objectives during consent process (Methods 2.6)
**8. Interviewer characteristics**	Researchers are nurses and academics with interest in patient decision-making and cardiovascular care (Author Information)

**Domain 2: Study Design**	
**9. Methodological orientation and theory**	Conventional content analysis, inductive approach (Methods 2.0, 2.4; Hsieh & Shannon, 2005; Erlingsson & Brysiewicz, 2017)
**10. Sampling**	Purposive sampling to ensure diversity in gender, age, education, and ethnicity (Methods 2.1)
**11. Method of approach**	Face-to-face recruitment at Bushehr Heart Hospital, with informed consent (Methods 2.3, 2.6)
**12. Sample size**	18 participants (11 patients, 2 family members, 2 nurses, 3 physicians) with 24 interviews (Methods 2.1)
**13. Non-participation**	No participants refused; some excluded due to medical emergencies or withdrawal (Methods 2.2)
**14. Setting of data collection**	Private hospital rooms for patients, educational spaces for staff (Methods 2.3)
**15. Presence of non-participants**	No non-participants present during interviews to ensure privacy (Methods 2.3)
**16. Description of sample**	10 men, 8 women; aged 41–78; diverse education and ethnicity (Persian, Arab, others) (Methods 2.1; Table 1)
**17. Interview guide**	Semi-structured guide with open-ended questions and probes; provided in Appendix A (Methods 2.3)
**18. Repeat interviews**	Six follow-up interviews conducted to clarify ambiguities (Methods 2.3)
**19. Audio/visual recording**	All interviews audio-recorded with participant consent (Methods 2.3, 2.6)
**20. Field notes**	Detailed notes taken during and after interviews to support analysis (Methods 2.5)
**21. Duration**	Interviews lasted 35–50 min (Methods 2.3)
**22. Data saturation**	Saturation achieved after 24 interviews, with no new categories emerging (Methods 2.1)
**23. Transcripts returned**	Participants could access transcripts upon request, as part of consent process (Methods 2.6)

**Domain 3: Analysis and Findings**	
**24. Number of data coders**	Multi-member team, including nurses and researchers (Methods 2.4)
**25. Description of the coding tree**	Codes grouped into 24 subcategories and 8 categories (e.g., Trust in Healthcare Providers, Treatment Transparency, and Infrastructure) (Methods 2.4; Table 2)
**26. Derivation of categories**	Categories derived inductively from meaning units, with example: “doctor explained clearly” → subcategory → category (Methods 2.4)
**27. Software**	MAXQDA20 used for coding and analysis (Methods 2.4)
**28. Participant checking**	Member-checking with five participants to verify categories (Methods 2.5)
**29. Quotations presented**	Direct quotes included with participant identifiers (e.g., P12) to illustrate categories (Results)
**30. Data and findings consistent**	Findings (8 categories) align with raw data, supported by quotes and audit trails (Methods 2.4, 2.5; Results)
**31. Clarity of major categories**	Eight main categories clearly presented as facilitators and barriers (Results; Table 2)
**32. Clarity of minor categories**	24 subcategories clearly linked to categories (e.g., transparent communication under Trust) (Methods 2.4; Table 2)
